# Supplementary material for: Dose-rate coefficients for external exposure to radionuclides uniformly distributed in soil to an infinite depth
Source: PLoS One. 2024 Sep 26;19(9):e0310552. doi: 10.1371/journal.pone.0310552 (PMC11426535; doi:10.1371/journal.pone.0310552)
Supplement: Attachment — Submitted filename: Response to Reviewers.docx [file pone.0310552.s014.docx]

**Response to Editor and Reviewers**

We wish to appreciate the reviewers' careful reading and insightful comments, which have helped us to significantly improve our manuscript. We have rewritten our manuscript by reflecting the reviewers' comments. Our responses to each comment are described below in blue.

**Journal Requirements:
Please review your reference list to ensure that it is complete and correct. If you have cited papers that have been retracted, please include the rationale for doing so in the manuscript text, or remove these references and replace them with relevant current references. Any changes to the reference list should be mentioned in the rebuttal letter that accompanies your revised manuscript. If you need to cite a retracted article, indicate the article’s retracted status in the References list and also include a citation and full reference for the retraction notice.**

We have confirmed that the reference list is complete and correct.

**Additional Editor Comments:**

**Please address each reviewer in a point by point response and make changes to the manuscript accordingly.**

As described below, we have revised the manuscript in response to the reviewers' comments and suggestions one by one. The revised text is highlighted in red in the marked-up copy of our manuscript.

In addition, some purely editorial, i.e. linguistic, edits have been made to make the manuscript better readable. None of these edits affect the results or conclusions of this paper. All changes are marked in blue in the marked-up copy of the revised manuscript.

**Reviewer #1:**

**1. The authors have conducted a limited comparison to prior work.
a) A short discussion is requested on the impact of specific organ dose contributions to the deviation comparing the ICRP reference and stylized phantoms (i.e., which organs most profoundly affected the effective dose calculation).
b) A selected comparison of specific age groups is also requested to determine the provenance of variability between phantom type vs. source generation methods**

We appreciate the reviewer's insightful suggestion. For further discussion regarding comparison with previous work, we revised Table 4 by adding data for a 10-year-old, 5-year-old, and newborn, as well as data for an additional radionuclide, i.e. ^50^V. We also added a respective paragraph at the Discussion section.

Under the present exposure conditions, and particularly for photon exposures, the equivalent dose rates of skin, muscle, and skeleton were slightly higher than those of other organs, but there are no organs or tissues that exhibit significantly higher dose rates.

The present results agree within 4% and 10% with those calculated for the voxel-type and stylized phantoms, respectively, except for the data of ^40^K by Bellamy et al. Regarding the electron component of ^40^K dose coefficients, please see response to comment No.2.

In response to your comment, we have compared our results with those from previous work for not only adults but also for 10-year-old, 5-year-old, and newborn. It was found that the deviations are not large for any specific age group. We have described this information in the main text.

Abstract:

“For verification, the results of the effective dose rates for the ^40^K, ^50^V, thorium, and uranium series, as well as ^137^Cs/^137m^Ba, were compared with those of previous studies and agreed within 10% for most cases.”

Page 18, Lines 317-334:

“**Table 4** provides a comparison of the effective dose-rate coefficients derived in the present study for adults, 10- and 5-year-old children, and newborns with values of previous studies. The data of the ^232^Th series, ^238^U series, and ^40^K were obtained from Petoussi-Henss et al. [4], who evaluated the dose rates for homogeneous distributions up to a depth of 100 cm in the soil using the ICRP voxel adult phantoms [17] and the Helmholtz Zentrum München (HMGU) voxel pediatric phantoms [25] in conjunction with the radiation-transport simulation code, EGSnrc [26]. The dose coefficients for ^137^Cs/^137m^Ba were derived by **Eq. (11)** from the respective data of ^137^Cs and ^137m^Ba from Bellamy et al. [3], who employed stylized hermaphrodite phantoms [27] and the simulation code, MCNP6 [28]. Their data were also used for the ^40^K and ^50^V.

The relative deviations between the data of the present work and those of Petoussi-Henss et al. [4], who used voxel phantoms, are less than 4%. The deviations to the data of Bellamy et al. [3], who used stylized hermaphrodite phantoms for the calculations, were found to be within 10% for ^137^Cs/^137m^Ba and ^50^V; note that for these nuclides, photons play a dominant role in the dose contribution. The equivalent dose rates of skin, muscle, and skeleton were slightly higher than those of the other organs and tissues; however, there are no organs or tissues that exhibit markedly higher dose rates than other organs. It was also found that the deviations between the present and previous results do not vary significantly among age groups.”

Table 4:

**“****Table 4 Ratio of the effective dose-rate coefficients reported by Petoussi-Henss et al. [4] and Bellamy et al. [3] for adults, 10- and 5-year-old children, and newborns to those obtained in the present work.** Here, 10-y and 5-y indicate 10- and 5-year-old children, respectively.

|  | Ratio of effective dose-rate coefficient | | | |
| --- | --- | --- | --- | --- |
| Nuclide | Adult | 10-y | 5-y | Newborn |
| ^232^Th series | 1.04^a^ | 1.00^a^ | 1.01^a^ | 1.03^a^ |
| ^238^U series | 1.04^a^ | 1.01^a^ | 1.00^a^ | 1.03^a^ |
| ^137^Cs/^137m^Ba | 1.08^b^ | 1.08^b^ | 1.05^b^ | 1.07^b^ |
| ^40^K | 1.04^a^ | 1.01^a^ | 1.00^a^ | 1.02^a^ |
|  | 1.27^b^ | 1.29^b^ | 1.25^b^ | 1.26^b^ |
| ^50^V | 1.07^b^ | 1.08^b^ | 1.05^b^ | 1.06^b^ |

^a^Derived from the data of Petoussi-Henss et al. [4].

^b^Derived from the data of Bellamy et al. [3].”

**2. The authors are requested to cite the original methods for the tabulation of the infinite soil dose coefficients, with discussion of comparison of methodology (beyond phantom) regarding source terms (e.g., comparison of bremsstrahlung calculated from both studies) from DOI:https://doi.org/10.1007/s00411-017-0692-7**

According to the reviewer’s comment, we have cited the proposed paper at Ref.[2]. Please note that the data presented at Ref.[3] are based on the methodology of Ref.[2]. Therefore, in the discussion, we compared the present results with those of Ref.[3]. For the simulation of photon and bremsstrahlung radiation, the approach used in the present work is the same as for Ref.[3]; and the deviation for nuclides emitting these radiations was found to be less than 10%. However, the methodology for estimating the electron skin dose is quite different. While we used a Monte-Carlo technique for both electron and photon transport, the authors of Ref.[3] used an electron scaled point-kernel approximation and geometrical reduction factors. We added the respective discussion in the main text.

Page 19, Lines 335-345:

“For ^40^K which releases a beta-minus particle per nuclear transformation with a branching fraction of 0.891, the deviations of the present results to the data of Bellamy et al. were up to approximately 30%. Both studies employed the same methodology [1] for the generation of bremsstrahlung by the electrons inside the soil and their contribution to organ equivalent dose rates. However, the methodology for the dose contribution to skin by the primary electrons released near the ground surface was quite different. Bellamy et al. used the point-kernel method [29] for calculating the electron skin dose, while the present work employed the results of radiation-transport simulations for electrons and photons. Since the deviations are less than 10% for nuclides for which photons are the major contributor to the dose rate, it can be concluded that the disagreement is due to the difference in methodology for estimating skin equivalent dose rate from primary electrons.”

Page 21, Lines 375-379:

“The results of certain typical nuclides were compared with those of previous studies. A good agreement within 10% was observed, except for ^40^K which undergoes beta-minus decay with branching fraction of 0.891. The present data for ^40^K, which were derived from the results of explicit electron and photon transport simulations, exhibited approximately 30% lower values than those of previous calculation [3] employing the point-kernel method for the estimation of skin equivalent dose rate by electrons.”

**3. The authors are requested to note the convergence criteria (e.g., 5% relative uncertainty) for the organs and tissue from the Monte Carlo simulation.**

According to the reviewer’s comment, we added text about the relative uncertainty of the Monte Carlo simulations of ICRP Publication 144.

Page 7, Lines 109-112:

“The combined relative uncertainties given as one standard deviation from both Step 1 and Step 2 simulations were less than 1% and 10%, respectively, for the air kerma rates and the equivalent dose rates of most organs and tissues.”

**4. While the authors have listed prior work having addressed soil dose coefficients, while only addressing surface soil, the following work might be cited as another work that employs the discussed method: https://doi.org/10.1007/s00411-019-00812-2**

According to the reviewer’s suggestion, we have cited the paper as Ref. [6] in Introduction. In addition, we have added a description of the two-step calculation method also used in Ref. [6] in Materials and methods.

Page 3, Lines 22-23:

“Nuclide-specific dose-rate coefficients have been evaluated to assess doses from external sources of radionuclides distributed in the environment [1-6].”

Page 5, Lines 71-81:

“Radiation-transport simulations starting from widely distributed radiation sources in the environment require considerable computing time to achieve sufficient statistical accuracy of the radiation field at its center. It is inefficient to repeat the time-consuming simulation for every sex- and age-specific phantom. Therefore, in the methodology adopted for the work of ICRP Publication 144 and other similar studies [1-6], the radiation-transport simulation was divided into two steps. (1) Simulation of the environmental radiation fields for monoenergetic photon and electron sources on and in the soil and (2) simulation of the doses absorbed by organs and tissues in the human body, i.e., reference phantoms exposed to environmental radiation. Therefore, the environmental radiation field obtained in Step 1 was used to the Step 2 simulation for the radiation transport in the phantoms.

In Step 1, planar sources of monoenergetic photons...”

**Reviewer #2:**

**In the introduction section you state, “Exposure to NORM poses no real risk of a radiological emergency leading to tissue reactions or immediate danger to life.” Additionally, throughout the article you again refer to the risk. Please further expand on the risk associated with NORM. It would be helpful for the reader to have a comparison to other radiation sources or other non-radiation hazards.**

Thank you for pointing this issue out. A risk associated with NORM varies depending on its radioactivity concentration and cannot be simply compared to risks associated with other radiation sources, but in most cases, the radioactivity concentration of NORM is not high enough to cause tissue reactions. As you pointed out, the risk associated with NORM industries is a multi-hazard situation and the radiological risk is generally not dominant. For this issue, an integrated and graded approach is recommended for the protection of workers, the public and the environment to complement and optimize the protection strategy already in place or planned to manage other hazards.

Considering the above discussion, we have revised and added text in the Introduction and Conclusions. Please note that the main subject of this paper is to provide dose-rate coefficients for radionuclides uniformly distributed in soil, which can contribute to the risk assessment of radiological hazard due to the prolonged exposures under the integrated approach. Therefore, we consider the discussion of quantitative risk assessment to be outside the scope of this paper and is not addressed here.

Page 4, Lines 48-53:

“Exposure to NORM poses no real risk of a radiological emergency leading to tissue reactions or immediate danger to life because its radioactivity concentration is not high [11]. However, NORM is widely distributed and causes prolonged exposures. Actions against the prolonged exposures of workers and the public are within the scope of the radiation protection system where the principles of justification and optimization should apply [12].”

Pages 21-22, Lines 386-389:

“In addition, the data could contribute to a risk assessment and management for the use of waste from NORM industries by considering both non-radiological and radiological hazards.”

**In the results and discussion section, “The age-dependent effective dose rates were higher for the younger age groups because children with shorter stature have organs and tissues closer to the soil, i.e., to the source.” Younger age groups have a higher sensitivity to radiation. Please address radiation sensitivity varies between an adult and a child. Based on your results, the risk associated to all age groups appears to be negligible.**

In this paper, we provided age-dependent dose-rate coefficients for effective dose as defined in the current ICRP Recommendations. The effective dose is used for radiological protection purposes and is obtained by multiplying the equivalent doses in tissues and organs by the weighting factors which reflect the radiosensitivity of those organs. In the current Recommendations, the ICRP states the following; “*(33) In view of the uncertainties surrounding the values of tissue weighting factors and the estimate of detriment, the Commission considers it appropriate for radiological protection purposes to use age- and sex-averaged tissue weighting factors and numerical risk estimates. The system of protection is sufficiently robust to achieve adequate protection for both sexes. Moreover, this obviates the requirement for sex- and age-specific radiological protection criteria which could prove unnecessarily discriminatory.*” (ICRP Publication 103, 2007).

Indeed, for the purposes of retrospective evaluation of radiation-related risks, it would be appropriate to use age-specific tissue weighting factors. However, the dose-rate coefficients presented in this paper are meant to be used for dose estimation for radiological protection purposes of the public living on a soil containing radionuclides. In the database of ICRP Publication 144, which was used to derive the present dose-rate coefficients, the age-dependent effective doses were computed with the same set of the tissue weighting factors for all age groups as per the current ICRP definition. Reflecting the above discussion, we have added the text in the Materials and methods.

Page 7, Lines 104-108:

“Effective dose rates were obtained based on the definition of the current ICRP system of radiological protection described in the ICRP 2007 Recommendations [8]. Therefore, the same set of tissue weighting factors was used in their computation for all age groups [7], and the differences in the effective dose rates as a function of age are only due to the anatomical differences in the phantoms.”

We wish to thank the reviewers again for his or her valuable comments. We hope that this revised manuscript will be accepted.
